# Supplementary material for: The yeast peroxisomal proteome at absolute quantitative scale
Source: Histochem Cell Biol. 2026 Feb 13;164(1):8. doi: 10.1007/s00418-026-02458-w (PMC12904895; doi:10.1007/s00418-026-02458-w)
Supplement: Supplementary file 1 — Supplementary file1 (PDF 3313 KB) [file 418_2026_2458_MOESM1_ESM.pdf]

The yeast peroxisomal proteome at absolute quantitative scale

Hirak Das[1*], Silke Oeljeklaus[1*], Renate Maier[1], Julian Bender[1], Bettina Warscheid[1]

[1]Faculty of Chemistry and Pharmacy, Biochemistry II, Theodor Boveri-Institute, Biocenter, University of Würzburg, 97094 Würzburg, Germany

*contributed equally to this publication

Corresponding author:
Bettina Warscheid (bettina.warscheid@uni-wuerzburg.de)

Supplementary information contents:

Supplementary Fig. S1
Supplementary Fig. S2
Supplementary Fig. S3
Legend to Supplementary Table S1
Legend to Supplementary Table S2
Legend to Supplementary Table S3

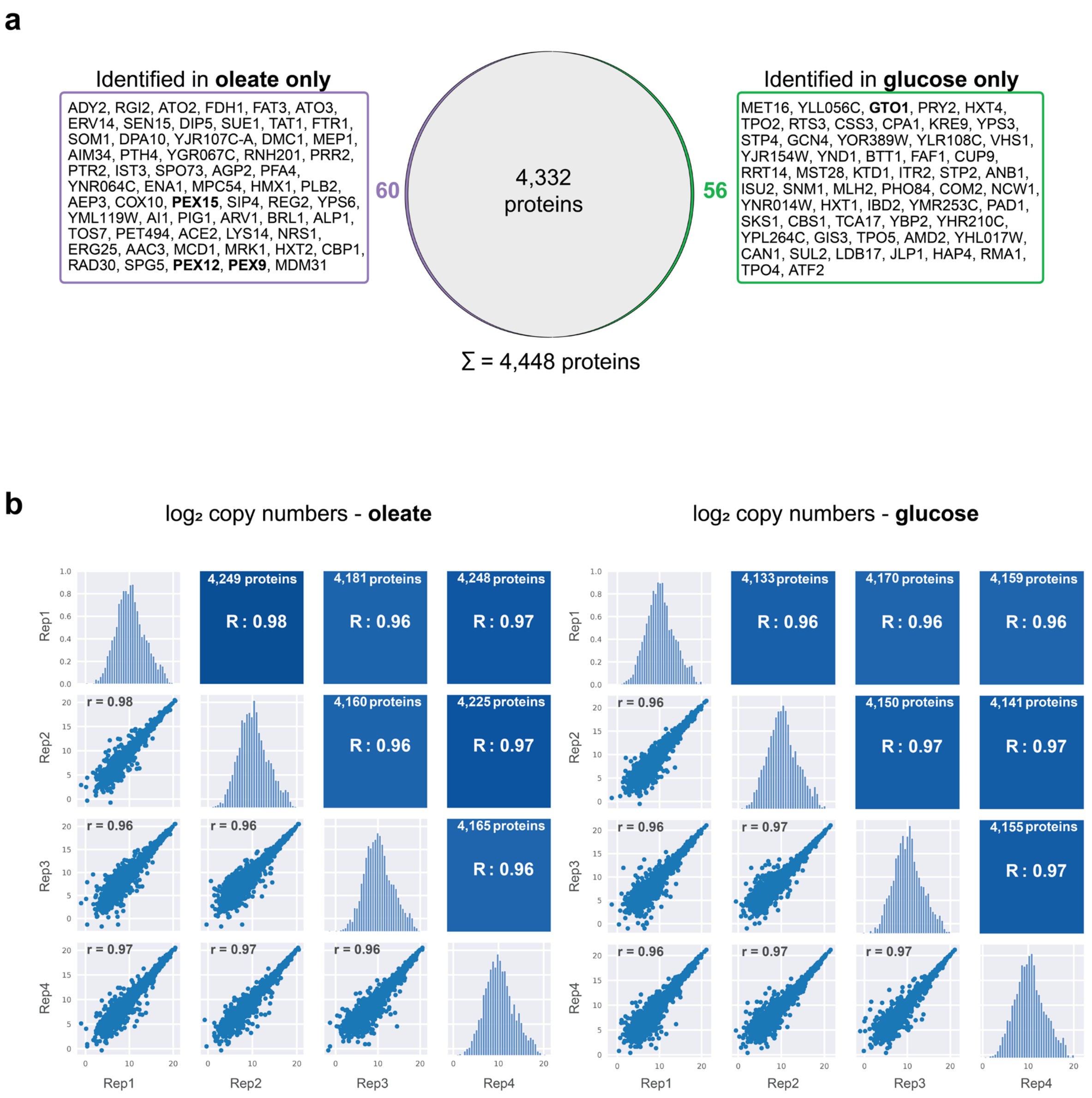

Supplementary Fig. S1 | Evaluation of cellular protein copy number estimation

Related to figure 1. **a.** Overlap of proteins identified in oleate- and glucose-grown cells. Proteins identified only under one of the conditions are listed; peroxisomal proteins are marked in bold. **b.** Multiscatterplot showing the reproducibility of protein copy numbers (log_2-transformed values) determined for individual replicates (Rep) of oleate- (left) and glucose-grown (right) cells (n=4 replicates each).

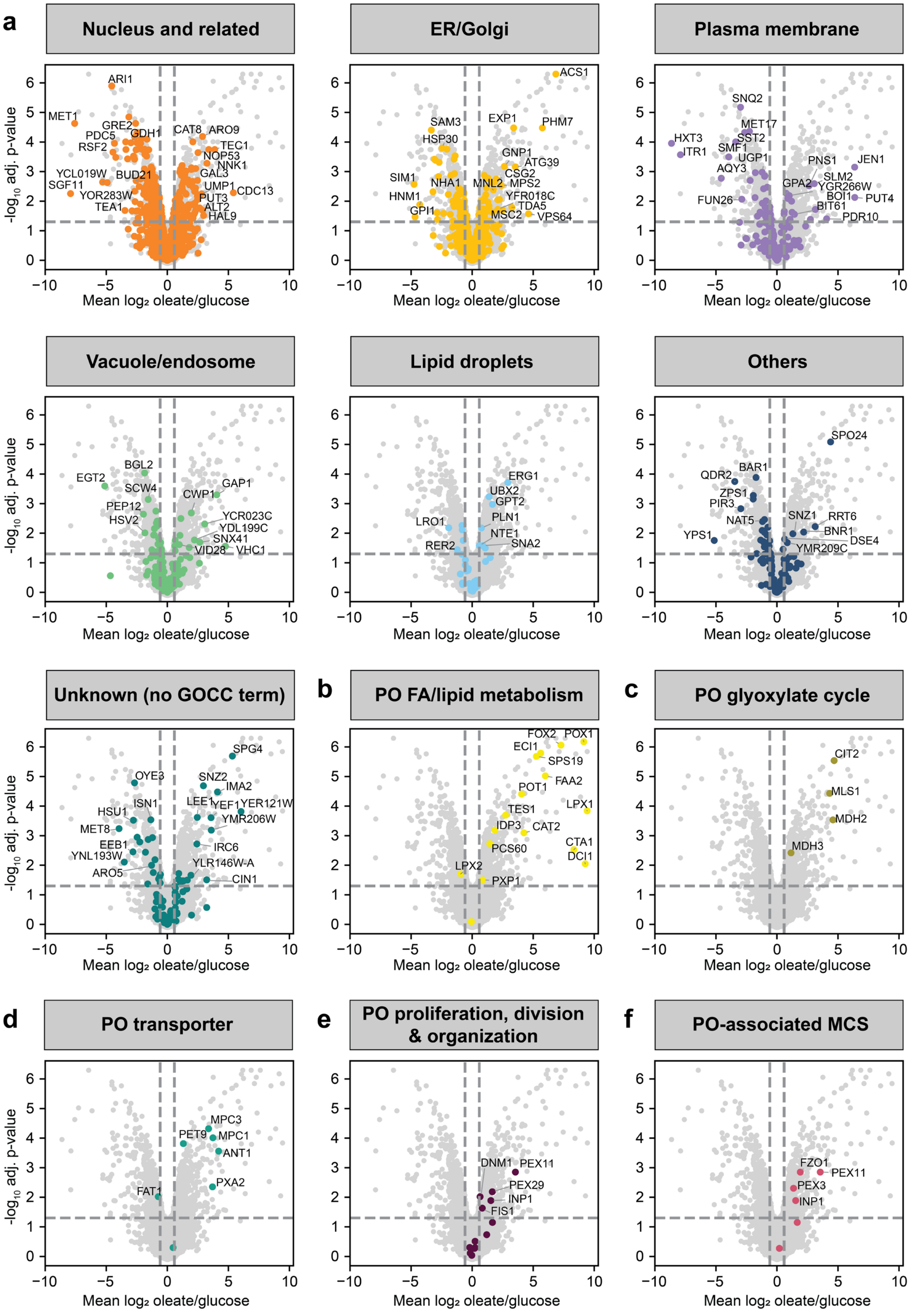
a
Nucleus and related
ARI1
MET1
GRE2
PDC5
GDH1
RSF2
CAT8
ARO9
TEC1
NOP53
NNK1
YCL019W
BUD21
GAL3
SGF11
UMP1
CDC13
YOR283W
PUT3
TEA1
ALT2
HAL9
ER/Golgi
ACS1
SAM3
EXP1
PHM7
HSP30
GNP1
ATG39
SIM1
CSG2
NHA1
MNL2
MPS2
HNM1
YFR018C
TDA5
GPI1
MSC2
VPS64
Plasma membrane
SNQ2
MET17
HXT3
SST2
SMF1
ITR1
UGP1
JEN1
PNS1
AQY3
SLM2
GPA2
YGR266W
FUN26
BOI1
PUT4
BIT61
PDR10
Vacuole/endosome
BGL2
EGT2
GAP1
SCW4
CWP1
PEP12
HSV2
YCR023C
YDL199C
SNX41
VID28
VHC1
Lipid droplets
ERG1
UBX2
GPT2
PLN1
LRO1
NTE1
RER2
SNA2
Others
SPO24
BAR1
QDR2
ZPS1
PIR3
NAT5
SNZ1
RRT6
YPS1
BNR1
DSE4
YMR209C
Unknown (no GOCC term)
SPG4
OYE3
SNZ2
IMA2
ISN1
LEE1
YEF1
YER121W
HSU1
YMR206W
MET8
EEB1
IRC6
YNL193W
YLR146W-A
ARO5
CIN1
b
PO FA/lipid metabolism
FOX2
POX1
ECI1
SPS19
FAA2
POT1
LPX1
TES1
IDP3
CAT2
CTA1
PCS60
DCI1
LPX2
PXP1
c
PO glyoxylate cycle
CIT2
MLS1
MDH2
MDH3
d
PO transporter
MPC3
PET9
MPC1
ANT1
PXA2
FAT1
e
PO proliferation, division & organization
DNM1
PEX11
PEX29
INP1
FIS1
f
PO-associated MCS
FZO1
PEX11
PEX3
INP1
-log10 adj. p-value
Mean log2 oleate/glucose
−10
−5
0
5
10
0
1
2
3
4
5
6

Supplementary Fig. S2 | Carbon source-dependent differences in the abundance of proteins of different subcellular localization and associated with different peroxiso-mal processes

Related to figure 2. **a.** Same plot as shown in Figure **2a** highlighting proteins of the indicated subcellular localization (**a**) and involved in different peroxisomal processes (**b – f**) as indicated. PO, peroxisomal; FA, fatty acid; MCS, membrane contact sites.

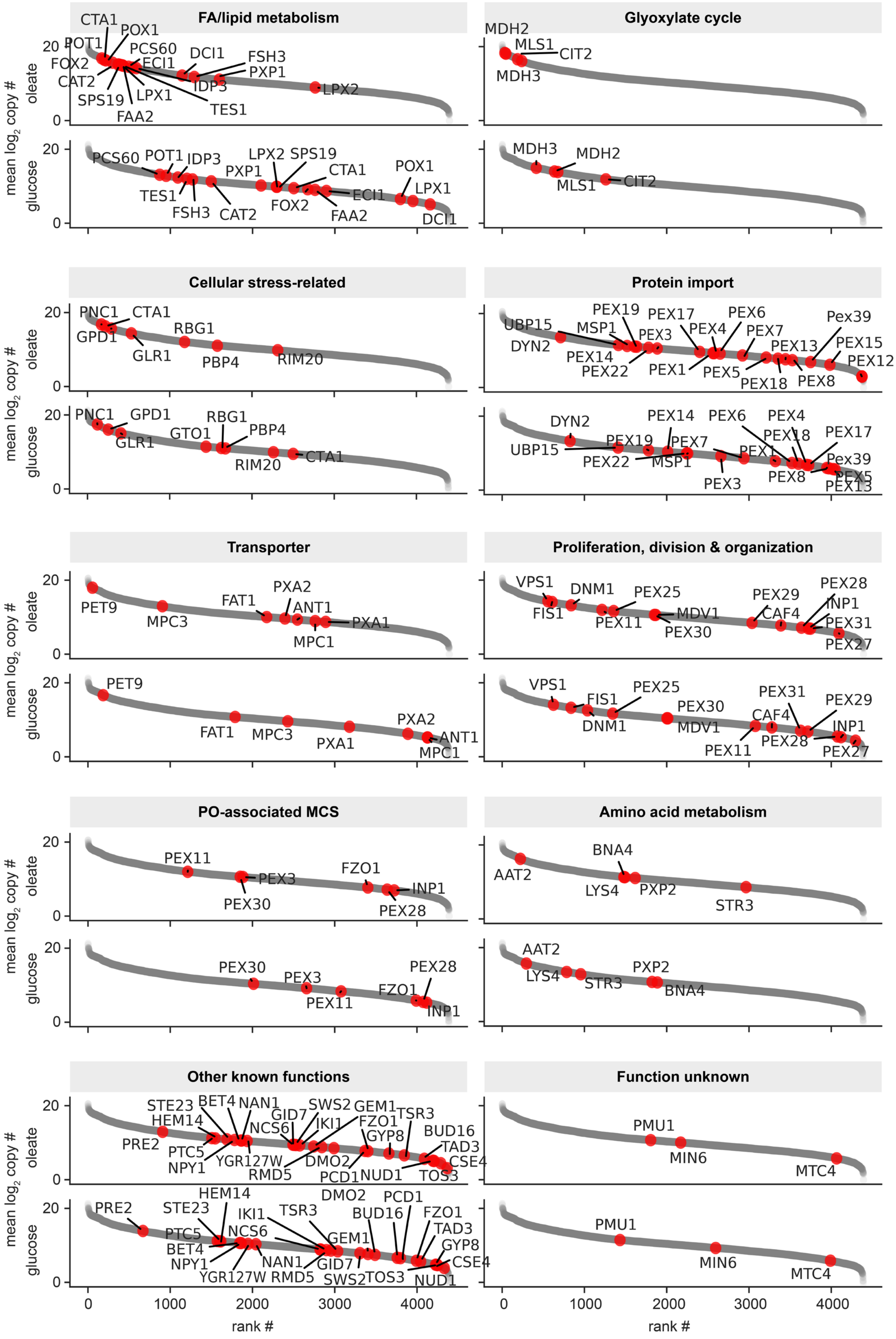
FA/lipid metabolism
CTA1
POX1
POT1
PCS60
DCI1
FSH3
FOX2
ECI1
PXP1
CAT2
IDP3
LPX2
SPS19
LPX1
TES1
FAA2
PCS60 POT1 IDP3
LPX2 SPS19
PXP1
CTA1
POX1
LPX1
TES1
ECI1
FSH3
CAT2
FOX2
FAA2
DCI1
Glyoxylate cycle
MDH2
MLS1
CIT2
MDH3
MDH3
MDH2
MLS1
CIT2
Cellular stress-related
PNC1
CTA1
RBG1
GPD1
GLR1
PBP4
RIM20
PNC1
GPD1
RBG1
GTO1
PBP4
GLR1
RIM20
CTA1
Protein import
PEX19 PEX17 PEX6
Pex39
UBP15
MSP1
PEX4
PEX7
PEX3
PEX13
PEX15
DYN2
PEX14
PEX12
PEX22
PEX1
PEX5
PEX18
PEX8
DYN2
PEX14 PEX6 PEX4
PEX18
PEX17
PEX19
PEX7
UBP15
PEX1
Pex39
PEX22
MSP1
PEX5
PEX3
PEX8
PEX13
Transporter
PXA2
FAT1
ANT1
PET9
MPC3
PXA1
MPC1
PET9
PXA2
ANT1
FAT1
MPC3
PXA1
MPC1
Proliferation, division & organization
VPS1
DNM1
PEX25
PEX28
PEX29
INP1
FIS1
CAF4
MDV1
PEX11
PEX31
PEX30
PEX27
VPS1
PEX25
FIS1
PEX31
PEX30
PEX29
CAF4
DNM1
MDV1
INP1
PEX28
PEX11
PEX27
PO-associated MCS
PEX11
FZO1
PEX3
INP1
PEX30
PEX28
PEX30
PEX28
PEX3
FZO1
PEX11
INP1
Amino acid metabolism
BNA4
AAT2
LYS4
PXP2
STR3
AAT2
LYS4
PXP2
STR3
BNA4
Other known functions
BET4 NAN1
STE23
SWS2 GEM1
GID7
TSR3
HEM14
NCS6
IKI1
FZO1
BUD16
PRE2
GYP8
PTC5
TAD3
NPY1
YGR127W
DMO2
CSE4
RMD5
PCD1 NUD1
TOS3
HEM14
DMO2
PCD1
PRE2
STE23
IKI1
TSR3
BUD16
FZO1
PTC5
NCS6
GEM1
TAD3
BET4
GYP8
NPY1
NAN1
GID7
CSE4
YGR127W RMD5
SWS2
TOS3
NUD1
Function unknown
PMU1
MIN6
MTC4
PMU1
MIN6
MTC4
mean $\log_2$ copy #
oleate
glucose
20
0
0
1000
2000
3000
4000
rank #

Supplementary Fig. S3 | Comparison of the absolute abundance of peroxisomal proteins in oleate- versus glucose-grown cells

Related to figure 3. Shown are the same rank plots as in **Fig. 3b** highlighting proteins associated with the indicated peroxisomal functions. For information about the exact protein copy numbers, see **Table 1**. Data shown refer to the complete peroxisomal proteome including peroxisomal core proteins and MLPs. FA, fatty acid; MCS, membrane contact sites; PO, peroxisomal.

Supplementary Table Legends

Supplementary Table S1 | Protein copy numbers per cell for *S. cerevisiae* grown in oleate- or glucose (xlsx file)
Protein copy numbers per cell were estimated based on MS1 intensities determined by MaxQuant from label-free LC-MS analyses of whole cell lysates from oleate- and glucose-grown cells (n = 4 each). The peroxisomal core proteome (column H) comprises all peroxins and proteins exclusively localized to peroxisomes. Classification as peroxisomal core or multi-localized protein (MLP, column I) is based on current knowledge and database annotations. SD, standard deviation; Glc, glucose; GOCC/GOBP/GOMF, Gene Ontology domain Cellular Component/Biological Process/Molecular Function.

Supplementary Table S2 | Proteins of the peroxisomal proteome (a) and peroxisomal proteins not identified in this study (b) (xlsx file)
a. The table includes the protein copy numbers and information about the classification as peroxisomal core or multi-localized protein (MLP), a second subcellular localization for MLPs, and functional classification of the peroxisomal proteins identified in our study, each based on current knowledge and database annotations. SD, standard deviation; Glc, glucose; PO, peroxisomal; FA, fatty acid. Please note that each protein was only assigned to one functional group, although proteins may have more than one function.
b. The table provides information about the occurrence of the peroxisomal proteins/peptides that were not identified in other MS studies (according to entries in the Yeast PeptideAtlas, https://peptideatlas.org/builds/yeast/) and membrane-association of the proteins.

Supplementary Table S3 | Results of GO term enrichment analyses (xlsx file)
The analysis was performed for the domains "Biological Process" and "Cellular Component" for proteins with significantly higher abundance in oleate- or glucose grown cells (adjusted p-value < 0.05; oleate/glucose ratio ≥ 1.5 or ≤ 0.6667).
